# Similarly oriented objects appear more numerous

**DOI:** 10.1167/jov.20.4.4

**Published:** 2020-04-09

**Authors:** Nicholas K. DeWind, Michael F. Bonner, Elizabeth M. Brannon

**Affiliations:** 1 Department of Psychology, University of Pennsylvania, Philadelphia, PA, USA; 2 Department of Cognitive Science, Johns Hopkins University, Baltimore, MD, USA

**Keywords:** numerosity, illusion, orientation coherence

## Abstract

Several non-numerical factors influence the numerical estimation of visual arrays, including the spacing of items and whether they are arranged randomly or symmetrically. Here we report a novel numerosity illusion we term the *coherence illusion*. When items in an array have a coherent orientation (all pointing in the same direction) they seem to be more numerous than when items are oriented randomly. Participants show parametric effects of orientation coherence in three distinct numerical judgment tasks. These findings are not predicted by any current model of numerical estimation. We discuss array entropy as a possible framework for explaining both the coherence illusion and the previously reported regular-random illusion.

## Introduction

Educated adults can precisely quantify a set by counting and using number words to denote cardinality. But people also directly perceive the approximate number of objects in a set (e.g., Dehaene, 1997). Educated adults share this number sense with primates (e.g., Brannon & Terrace, 1998), rodents (e.g., Meck & Church, 1983), birds (e.g., Honig & Stewart, 1989), and a wide variety of other animals (for review see Merritt, DeWind, & Brannon, 2012). The approximate number sense emerges early in human development (e.g., Izard, Sann, Spelke, & Streri, 2009) and is present in adults from societies that lack a verbal counting system (Pica, Lemer, Izard, & Dehaene, 2004). The approximate number sense is theorized to provide a foundation for symbolic mathematics (Feigenson, Dehaene, & Spelke, 2004), and the precision of approximate numerical discrimination has been found to be correlated with mathematical achievement in children (for review see Chen & Li, 2014).

Several non-numerical attributes of arrays have been noted to affect their perceived numerosity. For example, connecting elements in an array with a thin line decreases their perceived numerosity (Franconeri, Bemis, & Alvarez, 2009; He, Zhang, Zhou, & Chen, 2009). These authors propose that the perceived numerical decrease is due to object segmentation. Items that are part of a contiguous portion of space may be partially viewed as a single object. Two objects connected by a line are contiguously connected, and so an array composed of connected pairs is viewed as less numerous than the same array without the connections. Interestingly, this effect does not require an explicit spatial connection. Illusory contours connecting objects as well as statistical regularity in the color of neighboring objects is sufficient to create a more abstract “connection” between the objects and reduce perceived numerosity (Kirjakovski & Matsumoto, 2016; Zhao & Yu, 2016). Connectedness affects numerosity representations in extrastriate cortical areas and operates on signals associated with perceived numerosity rather than lower level representations in primary visual cortex. For example, connectedness affects numerosity adaptation and is detectable in neural responses to stimulus arrays after 150 ms in visual area V3, but not in lower cortical areas (Fornaciai, Cicchini, & Burr, 2016; Fornaciai & Park, 2018). Connectedness and grouping of objects also affects numerosity signals in the intraparietal areas (He, Zhou, Zhou, He, & Chen, 2015).

Another effect of array configuration on perceived numerosity is spacing; greater average spacing between the elements of a set increases their perceived number (DeWind, Adams, Platt, & Brannon, 2015; Gebuis & Reynvoet, 2012a), and regularly spaced elements seem to be more numerous than randomly spaced elements (Ginsburg, 1976). These effects may be two examples of the same phenomenon: that greater distance between array items increases perceived numerosity (Allïk & Tuulmets, 1991).

Orientation perception is fundamental to vision, and participants rapidly and readily discriminate orientation (Treisman & Gormican, 1988). Researchers have also found that participants can discriminate textures or arrays of objects based on their orientation variance, that is the representation of the second moment of a distribution of multiple orientations presented simultaneously (Dakin & Watt, 1997). Perception of orientation variance could be useful for detecting the boundaries between textures and may be a cue aiding in object segmentation. Indeed, many texture discrimination tasks can be performed by comparing orientation variances (Dakin, 1999). Orientation variance is susceptible to adaptation and adaptation to variance is experimentally distinguishable from adaptation to orientation itself, suggesting that orientation variance is directly encoded and represented by a unique mechanism (Norman, Heywood, & Kentridge, 2015).

**Figure 1.**
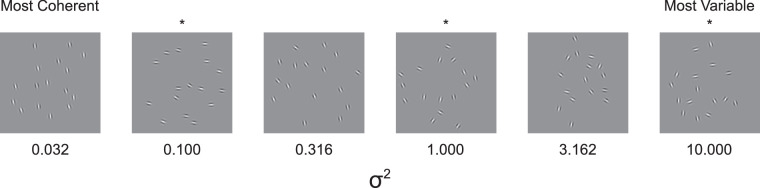
Example stimulus arrays. Example arrays at each orientation coherence. All arrays shown here contain 16 items. The values below the arrays indicate the variance in radians of the von Mises distribution from which orientations were drawn. Only the three coherences with asterisks above them were used in Experiments 1 and 3. All six coherences were used in Experiments 2 and 4.

Although Dakin et al. propose that numerosity is derived from texture and density perception (Dakin, Tibber, Greenwood, Kingdom, & Morgan, 2011; Morgan, Raphael, Tibber, & Dakin, 2014) other evidence suggests that texture perception and numerosity perception operate through different mechanisms (Anobile, Turi, Cicchini, & Burr, 2015; Burr, Anobile, & Arrighi, 2018). Orientation variance, however, may play an underlying role in both numerosity and texture perception. These findings lead to the possibility that numerosity discrimination is influenced by the degree of orientation variance or orientation coherence of array items. To test this hypothesis, we constructed arrays of oriented Gabor patches and experimentally manipulated the orientation of these patches. In particular, each array was constructed such that the patches coherently pointed in the same direction or chaotically pointed in arbitrary directions (Figure 1).

We did not have a strong hypothesis for how orientation coherence would affect numerosity perception. We broadly saw three possible outcomes based on the prior literature. First, orientation coherence might have no effect. There is no model of numerosity perception that makes a clear prediction on orientation coherence, and, in general, the identity and properties of individual objects in a visual array have not been reported to affect their perceived numerosity.

A second possibility was that orientation coherence might be analogous to the connectedness illusion, such that orienting objects in the same direction might generate an abstract connection between them, causing them to be partially viewed as a single object (Zhao & Yu, 2016). In other words, orienting the objects similarly might make them appear to be part of a larger whole, cause the array to be partially viewed as a single object, and so decrease the perceived numerosity.

Third, we thought that arrays with coherent orientation might seem to have a regularity, somewhat like the grid-like arrangements that cause the regular-random illusion. If a more abstract perception of regularity—as opposed to greater interitem distance—causes the overestimation of regularly spaced elements, then regularly oriented elements might be perceived as more numerous.

Over four experiments we used three distinct tasks to probe the effect of orientation coherence on perceived numerosity. Experiments 1 to 3 required an ordinal comparison between two arrays and Experiment 4 required participants to produce numerical estimates of single arrays. We controlled for differences in salience between the arrays and replicated our findings in four independent samples. Across all the experiments, we found that arrays with more coherent orientations were perceived as more numerous than arrays with variable orientations.

## General methods

All methods were approved by the Institutional Review Board at the University of Pennsylvania. Participants were compensated with course credit or paid 10 USD for a single 45-minute session. Stimuli were generated and displayed using custom MATLAB (The Mathworks Inc., Natick, MA) scripts and Psychtoolbox3 (Brainard, 1997; Kleiner et al., 2007; Pelli, 1997) extension running on a Dell personal computer. The monitor was 51 cm × 28.5 cm and had a resolution of 1920 pixels × 1080 pixels. Participants were seated approximately 56 cm away from the screen.

## Experiment 1

### Methods

#### Participants

Eleven participants were recruited from the University of Pennsylvania community (mean age, 20.4 years; 8 female and 3 male).

#### Stimuli and task

Arrays were composed of 8, 10, 11, 13, 16, 19, 23, 27, or 32 two-dimensional Gabor patches on a neutral gray background (Figure 1). The spatial wavelength (20 pixels per cycle), size (60 pixel diameter), and phase of the patches were constant across all arrays. The orientations of the patches were selected from the circular normal distribution (the von Mises distribution). The distribution wrapped around at a half-turn, because the patches are bilaterally symmetrical (an upward and downward oriented patch are identical). The mean of this distribution was a random orientation, and the variance was randomly selected from one of three levels: 0.1, 1.0, and 10.0 radians. A low variance resulted in all the orientations being coherent, and a large variance resulted in relatively diverse orientations.

On each trial, two arrays were presented simultaneously for 750 ms followed by the response cue. The numerical ratio of the two arrays ranged from 1:1 to 1:2 over five levels. The numerosities were chosen to be as close to evenly spaced on a logarithmic scale as possible, while rounding to whole numbers. As a result, the ratio between two numbers was approximately equal for a given difference between levels of numerosity. For example, 11:13 is a jump of one level and has a similar ratio to 23:27, also a jump of one level. The five ratios had equal probability of appearing on each trial. As a result of this scheme, the ratio between the arrays was not correlated with the numerical value of the arrays. Participants were instructed to indicate the array with the greater number of items using the arrow keys on a standard computer keyboard. Participants were instructed to respond as quickly and accurately as possible. Each participant completed 600 trials, approximately 120 trials at each numerical ratio, and each participant viewed 1,200 stimulus arrays, approximately 400 arrays at each orientation variance. These numbers are approximate because numerical ratio and orientation variance were both pseudorandomly generated. Numerical ratio and orientation variance were uncorrelated.

#### Analysis

We fit a generalized linear model to participants’ choices (left or right) with a probit link function and a binomial error distribution with regressors for the log of the numerical ratio of the two arrays and the log of the orientation variance ratio between the two arrays (Equation 1).

(1) $$\begin{array}{ccc} p\left(choose\;right\right)\; & = & \Phi (\beta_{Side}+\beta_{Num}log_{2}\left(r_{Num}\right)\\ & & +\;\beta_{\sigma^{2}}log_{10}\left(r_{\sigma^{2}}\right)) \end{array}$$

where Φ is the cumulative normal function, *p*(*choose* *right*) is the probability of choosing the stimulus presented on the right side of the screen; β_*Side*_ is the intercept, which indicates side bias; *r_Num_* is the ratio of the number of items on the right to the number of items on the left; $r_{\sigma^{2}}$ is the ratio of the variances of the von Mises distribution used to generate the orientations on the right divided by the variance of the von Mises used to generate the orientations on the left; and β_*Num*_ and $\beta_{\sigma^{2}}$ are the regression coefficients fit to the number and orientation variance regressors, respectively.

The bases of the logarithms for the ratios were selected because of the spacing of the ratios themselves. The numerical ratio varied from ½ to 2, and so the logarithm base two varied from –1 to 1. The variance ratio varied from 0.01 to 100 and so the logarithm base 10 varied from –2 to 2. The base of the logarithm affects the absolute magnitude of the fit coefficients, but not any of the statistics used to test our hypotheses.

Each participant's choice data were modeled separately. To test the hypothesis that orientation variance affected choices, we used a *t*-test to determine whether the orientation regression coefficients were significantly different from zero across participants. To confirm the results of the *t*-tests, we also fit a mixed-effects model. This model was identical to Equation 1, but was fit to all the data simultaneously with the inclusion of independent random-effects for the intercept and slopes grouped by participant.

**Figure 2.**
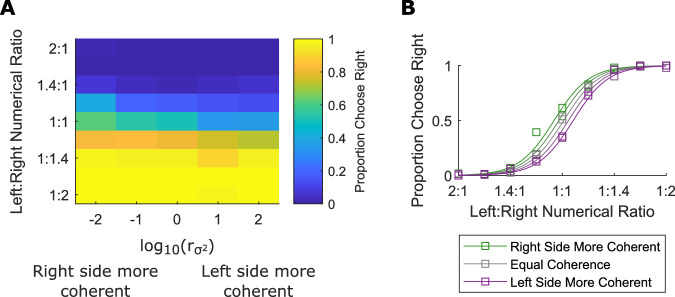
More coherent orientation increases estimates of numerosity. (A) The heat map shows the proportion of trials that participants choose the right (as opposed to left) stimulus at each numerical ratio and each difference in orientation coherence. Data pooled from all participants in Experiment 1. (B) The squares represent the same data as in (A), the lines are the best fit of the regression model in Equation 1 to the pooled data. The systematic shift of the numerical ratio curve across orientation coherence differences demonstrates the orientation coherence effect.

The fixed-effects model (Equation 1) was also fit to the pooled data from all participants. The results of this model fit were used to generate the psychometric curves plotted in Figure 2B; they represent the predicted probability of choosing the stimulus on the right at each of the five levels of orientation variance ratio over all numerical ratios. These curves were for visualization purposes and were not used for statistical hypothesis testing.

To provide a more intuitive representation of the magnitude of the coherence effect, we calculated the numerical ratio that would be necessary to effectively cancel the difference in perceived numerosity of the most and least coherent arrays. We used the participant average coefficient estimates from the generalized linear model, set $r_{\sigma^{2}}$to 0.01 and solved Equation 2 for *r_Num_*. This ratio was converted to a percentage to provide an easily interpretable expression.

(2) $$\beta_{\sigma^{2}}log_{10}\left(r_{\sigma^{2}}\right)=\beta_{Num}log_{2}\left(r_{Num}\right)$$

Accuracy was calculated as the proportion correct on all trials except those with a 1:1 ratio, for which there was no correct answer. These trials were, however, included for model fitting, because they were highly informative of the strength of the coherence effect.

### Results

All participants performed the task with well above chance accuracy (*M*: 93.4%, *SD*: 3.4%). Figure 2 shows the proportion of trials that participants selected the stimulus presented on the right side of the screen at different numerical and orientation variance ratios. The curved function demonstrates the numerical ratio effect. The separation of the curves based on orientation coherence ratio illustrates that increased coherence increased subjective numerical value. When the left side had greater variance (lower coherence) in orientation the right side was chosen more, and when the right side had great variance the left side was chosen more. To quantify these effects, we fit the regression model (Equation 1) to the choice data of each participant. We found a robust effect of numerical ratio on performance: the mean β_*Num*_ was significantly positive, *M*: 6.9, *SEM*: 0.49, t(10) = 14.0, *p* < 0.001. We also found a significant effect of orientation variance; the mean $\beta_{\sigma^{2}}$ was significantly negative, *M*: -0.3, *SEM*: 0.06, t(10) = –5.1, *p* < 0.001. We confirmed this finding using a mixed-effects model with participant treated as a random effect; we found a significant fixed-effect of numerical ratio, β_*Num*_ = 3.7, t(6597) = 14.5, *p* < 0.001, and variance ratio, $\beta_{\sigma^{2}}$ = -0.17, t(6597) = –5.5, *p* < 0.001. A negative coefficient indicates that more variable orientations seemed to be less numerous and more coherent orientations seemed to be more numerous. Ten of 11 participants had a negative $\beta_{\sigma^{2}}$ coefficient. On average, the most coherent arrays appeared 6.4% more numerous than the least coherent arrays, sufficient to make 15 coherent objects seem to be equal to 16 variable objects. When the numerosities presented were equal but one array was maximally coherent and the other was maximally variable, participants indicated that the more coherent array was greater 63% of the time. This is equivalent to the comparison of the most and least coherent arrays with asterisks in Figure 1.

We tested whether precision on the numerical comparison task was correlated with the effect of orientation coherence at the participant level. We found that there was no significant correlation between β_*Num*_ and $\beta_{\sigma^{2}}$ (r = –0.38, *p* = 0.24).

## Experiment 2

The results from Experiment 1 demonstrated an effect of orientation coherence on numerosity discrimination performance. We designed Experiment 2 to replicate the effect in a larger independent sample and include more coherence values. Because accuracy was so high in Experiment 1 and the orientation coherence effect could only be estimated when there was some variability in participants’ responses, we constructed a new stimulus set with finer gradations of orientation coherence and more difficult numerical ratios.

### Methods

Twenty-seven participants were recruited from the university community (mean age: 23.9 years; 18 female, 8 male, and 1 not reported). The methods were identical to Experiment 1 with the following exceptions. The 1:2 and 1.0:1.7 ratios were eliminated, making the 1.0:1.4 the easiest numerical ratio. The variance of the von Mises distribution were expanded to include 0.03, 0.10, 0.32, 1.00, 3.16, and 10.00 radians (Figure 1). Each participant saw approximate 200 trials at each of the three numerical ratios, and approximately 200 stimuli at each orientation variance.

### Results

**Figure 3.**
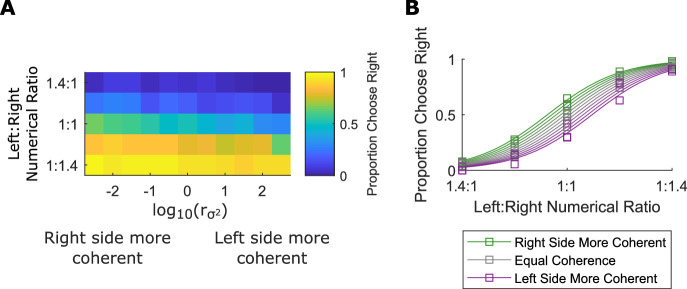
Replication of the orientation coherence effect with more levels of coherence in a larger sample. (A) The heat map shows the proportion of trials that participants choose the right stimulus broken out over all numerical and orientation coherence ratios. Data pooled from all participants in Experiment 2. (B) The squares represent the same data as in (A), the lines are the best fit of the regression model in Equation 1.

Despite the more difficult ratios, participants performed the task with well above chance accuracy (accuracy *M*: 87.3%, *SD*: 5.6%, *n* = 27). Figure 3 shows the proportion of trials that participants selected the right stimulus at different numerical and orientation coherence ratios. Again, the numerical ratio effect is illustrated by the curved functions in Figure 3 and the orientation coherence effects are apparent by the separation of the functions by coherence. We fit the regression model (Equation 1) to the choice data of each participant. As in Experiment 1, we found an effect of numerical ratio, β_*Num*_ *M*:  6.6, *SEM*: 0.37, t(26) = 17.9, *p* < 0.001, and an effect of orientation coherence/ variance, $\beta_{\sigma^{2}}$ *M*: –0.3, *SEM*: 0.03, t(26) = –10.8, *p* < 0.001. We confirmed this finding using a mixed-effects model with participant treated as a random effect; we found a significant fixed-effect of numerical ratio, β_*Num*_ = 3.7, t(16197) = 19.6, *p* < 0.001, and variance ratio $\beta_{\sigma^{2}}$ = –0.15, t(16197) = –10.8, *p* < 0.001. Twenty-six of 27 participants had a negative $\beta_{\sigma^{2}}$ coefficient. On average, the most coherent arrays seemed to be 7.6% more numerous than the least coherent arrays, sufficient to make 15 coherent objects appear equal to 16 variable objects. When the numerosities presented were equal but one array was maximally coherent and the other was maximally variable, participants indicated that the more coherent array was greater 68% of the time. This is equivalent to the comparison of the most and least coherent arrays in Figure 1.

We tested whether precision on the numerical comparison task was correlated with the effect of orientation coherence at the participant level. We found a significant negative correlation between β_*Num*_ and $\beta_{\sigma^{2}}$, meaning that greater precision on numerical comparison was associated with a stronger effect of coherence (r = –0.47, *p* = 0.014).

## Experiment 3

In Experiments 1 and 2, participants were instructed to select the array with the greater number of items. In both experiments when orientation coherence was greater, participants were more likely to select an array, which we took as evidence that they perceived coherent arrays as more numerous. An alternative explanation, however, is that coherent arrays are more salient and therefore more likely to be chosen regardless of their perceived numerosity. To rule out this possibility, we conducted a third experiment instructing participants to choose the array with fewer items. We reasoned that if higher coherence caused arrays to appear more numerous we would replicate the effect seen in Experiments 1 and 2 and the order of the colored curves shown in Figures 2B and 3B would be inverted in Experiment 3 to reflect the instruction to choose the less numerous array. Alternatively, if coherent arrays were merely more salient, we would expect participants to continue to choose the more coherent arrays, even when instructed to choose the less numerous array.

### Methods

Eleven participants were recruited from the university community (mean age: 20.4 years; 5 female and 6 male). The methods were identical to Experiment 1, except that the participants’ task was to choose the array with fewer items.

### Results

**Figure 4.**
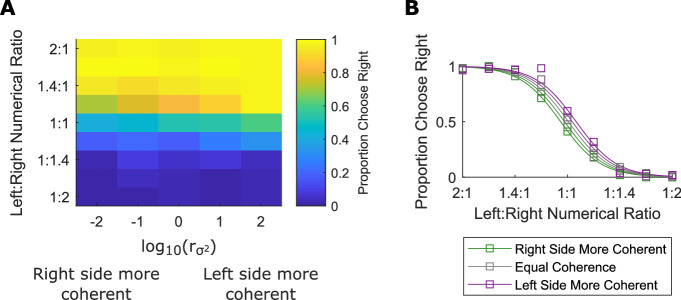
More variable (less coherent) arrays are perceived as less numerous. The same conventions as in Figures 2 and 3. Note that the participants in Experiment 3 are more likely to choose the array with fewer items and the more variable/less coherent orientations.

Participants performed the task with above chance accuracy (*M*: 92.6%, *SD*: 3.7%, *n* = 11). We fit the same regression model (Equation 1) to the choice data of each participant. As shown in Figure 4—consistent with the hypothesis that orientation coherence caused arrays to appear more numerous—we found that the sign of both the effect of numerical ratio, β_*Num*_ *M*:  –6.0, *SEM*: 0.49, t(10) = –12.3, *p* < 0.001, and the effect of orientation coherence/variance, $\beta_{\sigma^{2}}\;M:\;$0.2, *SEM*: 0.06, t(10) = 3.8, *p* = 0.003, was reversed compared with Experiments 1 and 2. We confirmed this finding using a mixed-effects model with participant treated as a random effect; we found a significant fixed-effect of numerical ratio, β_*Num*_ = –3.2, t(6597) = –13.0, *p* < 0.001, and variance ratio, $\beta_{\sigma^{2}}$ = 0.12, t(6597) = 3.9, *p* < 0.001. Nine of 11 participants had a positive $\beta_{\sigma^{2}}$ coefficient. On average, the most coherent arrays appeared 5.1% more numerous than the least coherent arrays, sufficient to make 19 coherent objects appear equal to 20 variable objects. When the numerosities presented were equal but one array was maximally coherent and the other was maximally variable, participants indicated that the least coherent array was less numerous 59% of the time.

We tested whether precision on the numerical comparison task was correlated with the effect of orientation coherence at the participant level. We found that there was no significant correlation between β_*Num*_ and $\beta_{\sigma^{2}}$ (r = –0.37, *p* = 0.27).

## Experiment 4

To test the generality of the effect we next explored whether the coherence illusion extends to numerical estimation judgments. Participants were presented with a single array and were instructed to estimate how many items it contained.

### Methods

Thirty-three participants were recruited from the university community (mean age: 24.3 years; 19 female and 14 male). The same stimuli were presented as in Experiment 2. However, a single array was presented for 750 ms. After each array appeared, participants were prompted to enter a numerical estimate using a standard keyboard. Participants were not informed of the true range of numerical values. Each participant performed 450 estimation trials. The numerosity and the orientation variance of the array were uncorrelated.

We fit the following regression model to the response data from each participant:

(3) $$\;log_{2}\left(Est\right)=\beta_{0}+\beta_{Num}log_{2}\left(Num\right)+\beta_{\sigma^{2}}log_{10}\left(\sigma^{2}\right)$$

where *Est* is the participant's estimate, *Num* is the real number of items, and σ^2^ is the variance of the orientations. β_0_, β_*Num*_, and $\beta_{\sigma^{2}}$ are the fit coefficients for the intercept, number of items, and orientation variance, respectively. To test the hypothesis that orientation variance affected choices, we used a *t*-test to determine whether the orientation regression coefficients were significantly different from zero across participants. To confirm the results of the *t*-tests, we also fit a mixed-effects model. This model was identical to Equation 3, but was fit to all the data simultaneously with the inclusion of independent random-effects for the intercept and slopes grouped by participant.

To examine the effect of coherence at each numerosity we fit Equation 4 to each participant's responses to each displayed numerosity. This model is identical to Equation 3, but has no term for numerosity.

(4) $$log_{2}\left(Est\right)=\beta_{0}+\beta_{\sigma^{2}}log_{10}\left(\sigma^{2}\right)$$

### Results

The mean, standard deviation, and coefficient of variation (defined as the standard deviation divided by the mean) of responses to each numerosity is presented in Table 1. Participants slightly overestimated arrays with between eight and 16 items and underestimated values above 16. Overall participants underestimated; the average (geometric mean) response was 93.9% of the displayed numerosity consistent with previous literature (Izard & Dehaene, 2008). With the exception of responses to eight items, which were more precise, the coefficient of variation did not systematically vary with numerosity, consistent with Weber's law as predicted by both a representation of numerosity on a logarithmic scale with constant variance or on a linear scale with scalar variance (Cantlon, Cordes, Libertus, & Brannon, 2009). The higher precision estimating eight items may have been due to rapid counting combined with true approximation (Mandler & Shebo, 1982).

**Table 1.** Summary of responses to each displayed numerosity.

Numerosity	8	10	11	13	16	19	23	27	32
Mean	8.5	10.6	11.7	13.6	16.2	18.3	20.3	22.8	25.1
SD	1.4	2.1	2.2	2.7	3.2	3.7	3.7	4.5	4.8
Coefficient of variation	0.157	0.193	0.186	0.195	0.195	0.200	0.183	0.194	0.189

*Note*: Mean, standard deviation, and coefficient of variation of responses to each of the nine displayed numerosities. Values were calculated for each participant and then averaged across participants (*n* = 33).

**Figure 5.**
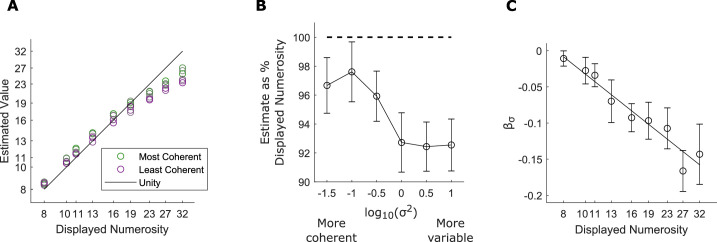
Orientation coherence increases numerosity estimates. (A) Average estimated value as a function of displayed numerosity at each coherence level. Estimates along the unity line are accurate. Data pooled from all participants in Experiment 4. (B) Estimates as a percentage of the displayed numerosity at each coherence level. Estimates at the dotted line would be accurate. (C) $\beta_{\sigma^{2}}$ fit separately for each numerosity. More negative $\beta_{\sigma^{2}}$indicates a larger coherence effect. Line is best fit of a mixed-effects linear regression with random intercept for each participant. Error bars indicate standard errors across participants (*n* = 33).

Figure 5 shows the average numerical estimates as a function of the number of items displayed and coherence level. Although on average participants underestimated, participants consistently estimated arrays with greater orientation coherence as more numerous than arrays with greater orientation variance. To quantify this effect, we fit the regression model in Equation 3 to each participant's data. We found that β_*Num*_ was significantly positive, indicating the participants were sensitive to the veridical number of items in the array, *M*: 0.75, *SEM*: 0.02, t(32) = 33.6, *p* < 0.001. We also found that $\beta_{\sigma^{2}}$ was significantly negative, indicating that participants viewed arrays with greater coherence as more numerous, *M*: –0.03, *SEM*: 0.004, t(32) = –9.1, *p* < 0.001. We confirmed this finding using a mixed-effects model with participant treated as a random effect; we found a significant fixed-effect of numerical ratio, β_*Num*_ = 8.3, t(14570) = 22.3, *p* < 0.001, and variance ratio, $\beta_{\sigma^{2}}$ = –0.07, t(14570) = –8.5, *p* < 0.001. Thirty-two of 33 participants had a negative $\beta_{\sigma^{2}}$.

To explore the interaction of numerical magnitude and the coherence effect, we separately estimated the coherence effect at each numerosity for each participant (Figure 5C). To test whether the coherence effect was significantly stronger ($\beta_{\sigma^{2}}$ was more negative) at larger numerosities, we fit a linear mixed-effects model to the data in Figure 5C and found a significant effect of numerosity (log_2_ of display numerosity) on $\beta_{\sigma^{2}}$, slope: –0.07; t(295) = –5.7, *p* < 0.001.

We tested whether precision on the numerical comparison task was correlated with the effect of orientation coherence at the participant level. We found that there was no significant correlation between coefficient of variation and $\beta_{\sigma^{2}}$ fit at the participant level (r = –0.13, *p* = 0.47).

## Discussion

Here we report a novel effect of orientation coherence on numerosity perception. Aligning the orientations of objects in an array increases their perceived numerosity, whereas increasing the orientation variance of an array systematically decreases perceived numerosity. The effect was robust over four independent samples and three types of numerosity judgment. Seventy-seven of 82 participants (94%) demonstrated susceptibility to the coherence illusion.

Orientation variance is a directly encoded visual percept and an important cue for discriminating texture and segmenting visual scenes (Dakin, 1999; Dakin & Watt, 1997; Norman et al., 2015). It is not surprising that such a feature would interact with numerosity perception; however, we did not have a strong prior hypothesis that variance or coherence would cause over- or underestimation. We broadly saw two possibilities—that coherence would be analogous to the connectedness illusion in that it would cause the array to appear as a unified whole thus decreasing perceived numerosity or that coherence would be analogous to the regular-random illusion and that array coherence would increase estimates of numerosity analogous to regularity.

Our results suggest that coherence and connectedness influence numerical estimations separately. One reason that orientation coherence may not have caused a connectedness illusion is that the connectedness illusion is induced by physical or abstract associations between pairs of objects within the arrays, whereas orientation coherence creates cohesion across the entire array. Future research could test whether creating pairs of nearby objects with similar orientation, while maintaining variable orientation across the entire array would cause numerosity to be perceived as even lower than in a completely randomly oriented array.

The regular-random illusion may be a result of differences in element spacing between regularly placed elements and randomly placed elements. For example, as more elements are placed into an array of fixed area, the variance in distances between elements decreases resulting in a positive correlation between numerosity and spacing regularity. Participants might consciously exploit this relationship, or unconsciously come to associate larger numerosity with regular spacing. Another proposal is “the occupancy model,” which suggests that regular-random illusion is just one example of a more general principle that greater spacing between items causes an increase in perceived numerosity (Allïk & Tuulmets, 1991). No explanation of the regular-random effect that relies entirely on element spacing can explain the orientation coherence effect reported here, because the arrays used here were equated for element size and spacing and only differed in orientation coherence. However, it is possible that orientation coherence affected perceived spacing, even if it did not affect actual spacing. Spaced out arrays are known to be perceived as more numerous (DeWind et al., 2015; Gebuis & Reynvoet, 2012b), and so orientation coherence may come to affect numerosity discrimination via an effect on perceived but not actual spacing. Relatedly, in the arrays used in these experiments, the Gabor patches were of constant size and were always drawn within a fixed array area. As a result, the total area covered by the patches and the density of the patches were correlated with the number of items in the arrays. Determining the effect of array coherence on numerical perception per se, rather than an effect on numerical perception that is mediated by non-numerical features will require experiments in which orientation coherence, size, and spacing are all systematically varied with respect to numerosity. Future research can also explore whether orientation variance affects the perception of density and area directly, and if so, whether those effects mediate the effect on numerosity perception.

Another framework that might be useful for understanding both the regular-random effect and the orientation coherence effect is the phenomenon of perceptual entropy. Entropy can be defined as a weighted average of the number of bits of information required to convey the orientations (or another feature) of an array. When a visual array is composed of objects of different categories, the category entropy of the array can be directly perceived (Young & Wasserman, 2001). Like approximate numerosity perception, entropy can be discriminated by animals as well as humans (Young & Wasserman, 1997). It is interesting to note that randomly spaced arrays have relatively high entropy because each element conveys a unique location. Similarly, randomly oriented arrays have relatively high entropy because each element conveys a unique orientation. Arrays that are arranged in a regular grid can be summarized with just the frequency and phase of the pattern and therefore can be conveyed with fewer bits of information. Similarly, perfectly coherent arrays can be described by a single orientation because all objects share the same orientation. Thus, an intriguing possibility is that both the regular-random illusion and the orientation coherence effect might be two facets of an underlying entropy mechanism, although the reason that low entropy inflates and high entropy deflates numerosity estimates is not immediately clear.

Consistent with the entropy account is our finding that the effect of orientation coherence increases with numerosity. Array entropy increases with the amount of feature variance in the items, but also with the number of items. An array with eight items encodes fewer bits of information than an array with 32 items when they both have the same variance. Thus, the increasing effect of orientation coherence observed when the number of items in the array was greater may be more properly understood as a constant effect of array entropy. Exploring this possibility will require a full mathematical treatment of entropy and further experiments in which entropy is systematically dissociated from variance and numerosity. Future research can also test the generality of the entropy proposal by testing the effect of other types of array coherence, such as color variance, on numerosity perception. Exploring the role of entropy in numerosity perception may help to relate the perception of numerosity to more fundamental principles of neural computation and information processing.

We also note that the effect of orientation coherence in Experiment 4 was nonlinear; the effect seemed to saturate at both the most coherent and most variable ends of the array variances we used. This was expected; to maximize our chance of discovering an effect, if it existed, we chose a range of variances spanning from arrays that seemed to be almost perfectly aligned to those that seemed to be completely random. Variance of the von Mises distribution is also susceptible to an artefact of circular distributions: as variance increases toward positive infinity, the distribution approaches a uniform sampling. As a result, there is no perceptual difference between an array with a variance of several radians and one with a variance of a several thousand radians, making a linear effect of statistical variance on any psychophysical measure impossible. Again, entropy may prove to have some explanatory power here, providing a measure of coherence with a more linear relationship to changes in numerosity perception. Homing in on the range of orientation coherences that have the largest differential effect on numerosity perception is an important future step, as is comparing that range to the perceptual sensitivity of orientation variance.

One piece of evidence against the entropy account is the finding that symmetrical arrays appear less numerous than asymmetrical arrays (Apthorp & Bell, 2015). Symmetrical arrays have lower entropy than their asymmetrical counterparts because the information sufficient to describe one-half of an asymmetrical array is sufficient to describe a whole symmetrical array. According to the entropy hypothesis developed here, symmetrical arrays should therefore be perceived as more rather than less numerous. However, the symmetry effect may be the result of a connectedness-like mechanism rather than an entropic mechanism, since the bilateral symmetry used in that study necessarily creates obvious pairs of array items.

We were interested in whether orientation coherence might serve as a heuristic for estimating numerosity similar to the way that some participants rely on item size or spacing in numerosity discrimination tasks (Gebuis & Reynvoet, 2012b). If it did, participants with poorer discrimination ability might have relied more on orientation coherence as an aid in discrimination. In Experiments 1, 3, and 4 we found no relationship between discrimination ability and the magnitude of the coherence effect, but in Experiment 2 we found a significant correlation between the numerosity and orientation variance model coefficients. However, the direction of this relationship does not support the idea that participants relied on orientation coherence more when they were less capable of discriminating numerosity. Rather, it was the participants who were better at discrimination who were more influenced by orientation coherence. This finding may have a simple explanation related to attentional lapses. Participants who occasionally guessed during the ordinal comparison task would have a lower estimated numerical discrimination ability and also a lower estimated orientation coherence effect. This is because, if participants made occasional responses unrelated to the stimuli they saw, the estimate of the influence of all stimulus features, numerosity and orientation coherence, would be attenuated. We also note that if there were a true relationship between the strength of the orientation coherence and the effect numerical acuity, we would also expect to see the relationship in Experiment 4 when participants were making estimates rather than choosing between two stimuli.

Finally, we note that it is unclear from our data whether orientation coherence increased perceived numerosity relative to a baseline estimate or instead increasing incoherence decreased perceived numerosity relative to a baseline estimate. A direct comparison of oriented and symmetric objects in a numerosity estimation task will resolve this question.

## Conclusion

Here we have shown across four independent samples and three types of numerosity judgment that similarly oriented objects are perceived as more numerous than randomly oriented objects. The coherence illusion is not predicted by previous models of approximate numerosity discrimination performance; however, future work should explore whether the orientation coherence illusion is one instance of a more general effect whereby low entropy arrays are perceived as more numerous than high entropy arrays. A full description of how non-numerical factors influence perceived numerosity should ultimately aid in uncovering the computational mechanisms by which approximate number is derived from visual input.

## References

[bib1] Allïk J., & Tuulmets T. (1991). Occupancy model of perceived numerosity. *Perception & Psychophysics,* 49(4), 303–314, 10.3758/BF03205986. 2030927

[bib2] Anobile G., Turi M., Cicchini G. M., & Burr D. C. (2015). Mechanisms for perception of numerosity or texture-density are governed by crowding-like effects. *Journal of Vision,* 15(5), 4–4, 10.1167/15.5.4. PMC4909146 26067522

[bib3] Apthorp D., & Bell J. (2015). Symmetry is less than meets the eye. *Current Biology,* 25(7), R267–R268, 10.1016/j.cub.2015.02.017. 25829006

[bib4] Brainard D. H. (1997). The Psychophysics Toolbox. *Spatial Vision,* 10(4), 433–436. 9176952

[bib5] Brannon E. M., & Terrace H. S. (1998). Ordering of the Numerosities 1 to 9 by Monkeys. *Science,* 282(5389), 746–749, 10.1126/science.282.5389.746. 9784133

[bib6] Burr D. C., Anobile G., & Arrighi R. (2018). Psychophysical evidence for the number sense. *Philosophical Transactions of the Royal Society B: Biological Sciences,* 373(1740), 20170045, 10.1098/rstb.2017.0045. PMC5784049 29292350

[bib7] Cantlon J. F., Cordes S., Libertus M. E., & Brannon E. M. (2009). Comment on “Log or linear? Distinct intuitions of the number scale in Western and Amazonian indigene cultures”. *Science,* 323(5910), 38b–38b. 19119201 10.1126/science.1164878 PMC3393850

[bib8] Chen Q., & Li J. (2014). Association between individual differences in non-symbolic number acuity and math performance: A meta-analysis. *Acta Psychologica,* 148, 163–172, 10.1016/j.actpsy.2014.01.016. 24583622

[bib9] Dakin S. C. (1999). Orientation variance as a quantifier of structure in texture. *Spatial Vision,* 12(1), 1–30. 10195386 10.1163/156856899x00012

[bib10] Dakin S. C., Tibber M. S., Greenwood J. A., Kingdom F. A. A., & Morgan M. J. (2011). A common visual metric for approximate number and density. *Proceedings of the National Academy of Sciences of the United States of America,* 108(49), 19552–19557, 10.1073/pnas.1113195108. 22106276 PMC3241748

[bib11] Dakin S. C., & Watt R. J. (1997). The computation of orientation statistics from visual texture. *Vision Research,* 37(22), 3181–3192, 10.1016/s0042-6989(97)00133-8. 9463699

[bib12] Dehaene S. (1997). *The number sense how the mind creates mathematics*. New York: Oxford University Press.

[bib13] DeWind N. K., Adams G. K., Platt M. L., & Brannon E. M. (2015). Modeling the approximate number system to quantify the contribution of visual stimulus features. *Cognition,* 142, 247–265, 10.1016/j.cognition.2015.05.016. 26056747 PMC4831213

[bib14] Feigenson L., Dehaene S., & Spelke E. (2004). Core systems of number. *Trends in Cognitive Sciences,* 8(7), 307–314, 10.1016/j.tics.2004.05.002. 15242690

[bib15] Fornaciai M., Cicchini G. M., & Burr D. C. (2016). Adaptation to number operates on perceived rather than physical numerosity. *Cognition,* 151, 63–67, 10.1016/j.cognition.2016.03.006. 26986745 PMC5040501

[bib16] Fornaciai M., & Park J. (2018). Early numerosity encoding in visual cortex is not sufficient for the representation of numerical magnitude. *Journal of Cognitive Neuroscience,* 30(12), 1788–1802, 10.1162/jocn_a_01320. 30063175

[bib17] Franconeri S. L., Bemis D. K., & Alvarez G. A. (2009). Number estimation relies on a set of segmented objects. *Cognition,* 113(1), 1–13, 10.1016/j.cognition.2009.07.002. 19647817

[bib18] Gebuis T., & Reynvoet B. (2012a). The interplay between nonsymbolic number and its continuous visual properties. *Journal of Experimental Psychology: General,* 141(4), 642–648, 10.1037/a0026218. 22082115

[bib19] Gebuis T., & Reynvoet B. (2012b). The role of visual information in numerosity estimation. *PLoS One,* 7(5), e37426, 10.1371/journal.pone.0037426. 22616007 PMC3355123

[bib20] Ginsburg N. (1976). Effect of item arrangement on perceived numerosity: Randomness vs regularity. *Perceptual and Motor Skills,* 42(43), 663–668. 10.2466/pms.1976.43.2.663 980664

[bib21] He L., Zhang J., Zhou T., & Chen L. (2009). Connectedness affects dot numerosity judgment: Implications for configural processing. *Psychonomic Bulletin & Review,* 16(3), 509–517, 10.3758/PBR.16.3.509. 19451377

[bib22] He L., Zhou K., Zhou T., He S., & Chen L. (2015). Topology-defined units in numerosity perception. *Proceedings of the National Academy of Sciences of the United State of America,* 112(41), E5647–E5655, 10.1073/pnas.1512408112. PMC4611617 26417075

[bib23] Honig W. K., & Stewart K. E. (1989). Discrimination of relative numerosity by pigeons. *Animal Learning & Behavior,* 17(2), 134–146.

[bib24] Izard V., & Dehaene S. (2008). Calibrating the mental number line. *Cognition,* 106(3), 1221–1247, 10.1016/j.cognition.2007.06.004. 17678639

[bib25] Izard V., Sann C., Spelke E. S., & Streri A. (2009). Newborn infants perceive abstract numbers. *Proceedings of the National Academy of Sciences of the United States of America,* 106(25), 10382–10385, 10.1073/pnas.0812142106. 19520833 PMC2700913

[bib26] Kirjakovski A., & Matsumoto E. (2016). Numerosity underestimation in sets with illusory contours. *Vision Research,* 122, 34–42, 10.1016/j.visres.2016.03.005. 27038561

[bib27] Kleiner M., Brainard D., Pelli D., Ingling A., Murray R., & Broussard C. (2007). What's new in Psychtoolbox-3. *Perception,* 36(14), 1–16.

[bib28] Mandler G., & Shebo B. J. (1982). Subitizing: An analysis of its component processes. *Journal of Experimental Psychology: General,* 111(1), 1–22, 10.1037/0096-3445.111.1.1. 6460833

[bib29] Meck W. H., & Church R. M. (1983). A mode control model of counting and timing processes. *Journal of Experimental Psychology: Animal Behavior Processes,* 9(3), 320–334, 10.1037/0097-7403.9.3.320. 6886634

[bib30] Merritt D. J., DeWind N. K., & Brannon E. M. (2012). Comparative cognition of number representation. *The Oxford Handbook of Comparative Cognition*. Oxford, UK: Oxford University Press, 10.1093/oxfordhb/9780195392661.013.0024.

[bib31] Morgan M. J., Raphael S., Tibber M. S., & Dakin S. C. (2014). A texture-processing model of the ‘visual sense of number’. *Proceedings of the Royal Society B: Biological Sciences,* 281(1790), 20141137, 10.1098/rspb.2014.1137. PMC4123707 25030988

[bib32] Norman L. J., Heywood C. A., & Kentridge R. W. (2015). Direct encoding of orientation variance in the visual system. *Journal of Vision,* 15(4), 3, 10.1167/15.4.3. 26067349

[bib33] Pelli D. G. (1997). The VideoToolbox software for visual psychophysics: Transforming numbers into movies. *Spatial Vision,* 10(4), 437–442. 9176953

[bib34] Pica P., Lemer C., Izard V., & Dehaene S. (2004). Exact and approximate arithmetic in an Amazonian indigene group. *Science,* 306(5695), 499–503. 15486303 10.1126/science.1102085

[bib35] Treisman A., & Gormican S. (1988). Feature analysis in early vision: Evidence from search asymmetries. *Psychological Review*, 95(2), 15–48. 3353475 10.1037/0033-295x.95.1.15

[bib36] Young M. E., & Wasserman E. A. (1997). Entropy detection by pigeons: Response to mixed visual displays after same–different discrimination training. *Journal of Experimental Psychology: Animal Behavior Processes,* 23(2), 157–170, 10.1037/0097-7403.23.2.157. 9095540

[bib37] Young M. E., & Wasserman E. A. (2001). Entropy and variability discrimination. *Journal of Experimental Psychology: Learning, Memory, and Cognition,* 27(1), 278–293, 10.1037/0278-7393.27.1.278. 11204103

[bib38] Zhao J., & Yu R. Q. (2016). Statistical regularities reduce perceived numerosity. *Cognition,* 146, 217–222, 10.1016/j.cognition.2015.09.018. 26451701

